# Case report: Rechallenge with EGFR–TKIs after immunotherapy in EGFR–mutated non–small cell lung cancer with leptomeningeal metastasis

**DOI:** 10.3389/fonc.2022.957661

**Published:** 2022-11-15

**Authors:** Chunfa Qian, Yuhai Zhang, Wanwan Cheng, Qingchao Zhang, Mengzhen Li, Shencun Fang

**Affiliations:** ^1^ Department of Neurosurgery, The Affiliated Brain Hospital of Nanjing Medical University, Nanjing, China; ^2^ Department of Respiratory Medicine, Nanjing Chest Hospital, The Affiliated Brain Hospital of Nanjing Medical University, Nanjing, China; ^3^ MyGene Diagnostics Co., Ltd., Guangzhou, China

**Keywords:** lung adenocarcinoma, EGFR-TKIs rechallenge, immune checkpoint inhibitor, the third generation EGFR-TKIs, leptomeningeal metastasis

## Abstract

Rechallenge of epidermal growth factor receptor-tyrosine kinase inhibitors (EGFR-TKIs) after PD-1 blockade failure was an effective therapy for non-small cell lung cancer (NSCLC) patients with resistance to EGFR-TKIs. The third-generation TKIs, like osimertinib and furmonertinib, can reach higher concentration in the cerebrospinal fluid (CSF) than other TKIs, and exhibit a beneficial effect in NSCLC patients with leptomeningeal metastases (LM) harboring sensitive EGFR mutation. Here, we report that two-stage IV pulmonary adenocarcinoma patients with LM harboring an EGFR L858R mutation benefit from the third-generation EGFR-TKIs rechallenge after immune checkpoint inhibitor (ICI) and anti-angiogenic agent combination therapy. Complete response (CR) to partial response (PR) of central nervous system (CNS) response was achieved immediately after the administration of furmonertinib and osimertinib. We conducted next-generation sequencing (NGS) and IHC to elucidate the evolution of driver mutations and the immune microenvironment. In conclusion, these two cases might provide a therapeutic strategy for further clinical practice. More research was needed to elucidate the resistance mechanisms and improve current treatment strategies in EGFR-mutated patients with LM.

## Introduction

Although patients with advanced NSCLC harboring drug-sensitive EGFR mutations benefit from the use of EGFR-TKIs, most of them progress within 12 months from the start of treatment due to acquired resistance (1). In addition, approximately 40% of EGFR-mutated NSCLC patients present with disease progression in the central nervous system (CNS), either as brain metastases (BM) or leptomeningeal metastases (LM) after initial EGFR-TKI treatment failure (2). LMs are associated with poor prognosis in NSCLC patients (3). Although underdiagnosed, about 10% of EGFR-mutated NSCLC patients experience LM during systemic TKI therapy, leading to dismal outcomes with a median survival of fewer than 6 months (4, 5). In clinical practice, many physicians frequently provide a chemotherapy or immunotherapy break followed by EGFR-TKI retreatment (3, 6). However, the optimal treatment for patients with EGFR-mutated NSCLC and LM that develops after EGFR-TKI therapy failure remains unclear. It is essential to explore the effectiveness of subsequent EGFR-TKI rechallenges after initial TKI failure in patients with NSCLC and LM.

In this study, we report two cases to evaluate the effectiveness of EGFR-TKIs rechallenge in EGFR-mutated NSCLC patients with LM after TKI failure and interspersed immunotherapy.

## Case report 1

**Figure 1:**
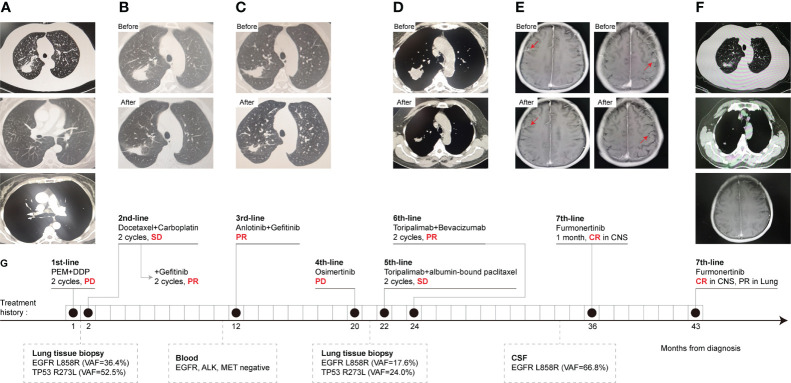
CT scans, treatment history, and gene mutation of case 1 at different clinical time points as shown. CT scans results. **(A)** Chest CT scans of right lung adenocarcinoma and lymph nodes before first-line treatment. **(B)** PR was observed after gefitinib was added to the combined chemotherapy, second-line treatment. **(C)** The change of lung lesion before and after third-line treatment, anlotinib plus gefitinib. **(D)** CT scans before and after two cycles of terriprizumab plus bevacizumab, sixth-line treatment. **(E)** MRI of the brain. CR of LM was obtained after 1 month of furmonertinib, seventh-line treatment. **(F)** The lung and CNS lesions were kept stable until recent follow-up in April 2022. **(G)** The timeline of Case 1 treatment history, follow-up diagnostic and gene test results. Numbers indicate the time from the diagnosis of NSCLC. CR, complete response; PR, partial response; SD, stable disease, PD, progressive disease; CSF, cerebrospinal fluid; CNS, central nervous system; VAF, variant allele frequency.

A 62-year-old Chinese female without a smoking history was admitted for a cough in September 2018. A computed tomography (CT) of the patient’s chest showed a mass in the right upper lung and diffuse micronodules in both lungs (**Figure 1A**). A needle biopsy of the mass revealed lung adenocarcinoma. Pemetrexed plus cisplatin were started as first line chemotherapy for two cycles until the disease progressed. CT examination showed that the right upper lung lesion was enlarged and the carcinoembryonic antigen (CEA) level was increased. Then docetaxel and carboplatin were chosen as the second-line treatments, and stable disease (SD) was achieved. Meanwhile, subsequent NGS of lung tissue identified the EGFR L858R mutation and the TP53 exon 8 mutation. PR was achieved after gefitinib (250 mg once daily) was added to the treatment for two cycles (**Figure 1B**). After 9 months, anlotinib (12 mg once daily) plus gefitinib (250 mg once daily) were initiated for pulmonary lesion progression, and no mutation was detected in plasma. PR was observed according to the CT scan (**Figure 1C**). Seven months later, re-examined CT suggested multiple metastases in both lungs, and the blood CEA was higher than before. Considering the progression of the disease, a pulmonary puncture was performed to search for drug-resistant genes, but the size of the puncture specimens was too small to conduct gene testing. For 1 month, empiric osimertinib was administered, but the disease still progressed. A lung biopsy was performed again on the right upper lung lesion and NGS was performed. The NGS results showed that EGFR L858R mutation, TP53 exon 8 missense mutation, and PD-L1 high expression (TPS = 60%), without EGFR T790M mutation. Toripalimab combined with albumin-bound paclitaxel was administered for two cycles. SD was achieved. But due to the intolerance of chemotherapy toxicity, the treatment regimen was changed to toripalimab plus bevacizumab. PR was achieved after two cycles of treatment (**Figure 1D**). Approximately 12 months later, she developed dizziness, headache, and vomiting and was diagnosed with LM based on cranial enhanced magnetic resonance imaging (MRI) and CSF cytological analysis. DNA sequencing of CSF and plasma specimens revealed an EGFR L858R mutation. The EGFR T790M mutation was undetectable. After 3 days of administration of furmonertinib (160 mg once daily), the neurological symptoms disappeared completely. After 1 month of EGFR-TKI rechallenge, CR of intracranial lesions was further confirmed (**Figure 1E**). The patient had received furmonertinib for more than 6 months, and no evidence of malignancy recurrence was found by brain MRI (**Figure 1F**). The treatment history and gene test results of this patient are presented in **Figure 1G**.

## Case report 2

**Figure 2:**
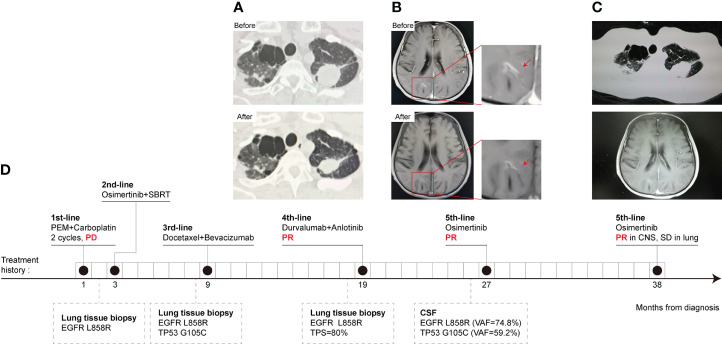
CT scans, treatment history, and gene mutation of Case 2 at different clinical time points as shown. **(A)** Chest CT scans. PR was observed after durvalumab plus anlotinib, fourth-line treatment. **(B)** MRI of the brain. A PR of LM was obtained after two cycles of osimertinib rechallenge. **(C)** The lung and CNS lesion kept stable till recent follow-up in April 2022. **(D)** The timeline of Case 2 treatment history, follow-up diagnostic and gene test results. The numbers indicate the time from the diagnosis of NSCLC.

A 49-year-old Chinese male with a smoking history was admitted to the hospital due to left lung adenocarcinoma with multiple bone metastases in January 2019. Then two cycles of pemetrexed plus carboplatin were initiated. SD was achieved in the pulmonary lesion. However, brain metastasis at the right frontoparietal junction was found by cranial enhanced MRI, and subsequent NGS of lung tissue identified an EGFR L858R mutation, without a T790M mutation. Then, the patient received second-line osimertinib treatment and cranial SBRT local radiotherapy (DT: 50 Gy/10 F) at the same time. Progression of lung lesions was observed by chest CT after 6 months. So docetaxel plus bevacizumab was started as the third-line treatment for 6 months. Subsequently, bevacizumab therapy was maintained for another 3 months. As pulmonary metastases and bone metastases progressed, another lung biopsy NGS testing revealed an EGFR L858R mutation and high PD-L1 expression (TPS = 80%). The EGFR T790M mutation was undetectable. Therefore, the patient commenced on four-line treatment with anlotinib and durvalumab. PR was observed after two cycles (**Figure 2A**). But the patient developed a headache, with vomiting and a static tremor 8 months later. Metastatic adenocarcinoma cells were observed by IHC and EGFR L858R, TP53, and KRAS amplifications were obtained by NGS in CSF samples. Based on contrast-enhanced MRI, LM was indicated. The osimertinib rechallenge significantly relieved the headache after one week. Moreover, PR of LM occurred after two cycles of treatment (**Figure 2B**). Finally, the osimertinib rechallenge was maintained for more than 11 months and the patient was still in close follow-up (**Figure 2C**). The treatment history and gene test results are presented in **Figure 2D**.

## Discussion

To the best of our knowledge, this is the first report to evaluate the efficacy of third-generation EGFR-TKI rechallenge for EGFR L858R mutated NSCLC patients with LM after immunotherapy. This report shows that EGFR-TKI rechallenge after ICI failure provides a prolonged PR in intracranial lesions for NSCLC patients with LM. Case 2 showed that rechallenge with a previously administered EGFR-TKI after the onset of LM may be an effective treatment strategy, not just switching to an un-administered EGFR-TKI like in Case 1. Besides, the use of CSF as a liquid biopsy specimen may facilitate precise diagnosis and personalized treatment for NSCLC with LM.

**Figure 3:**
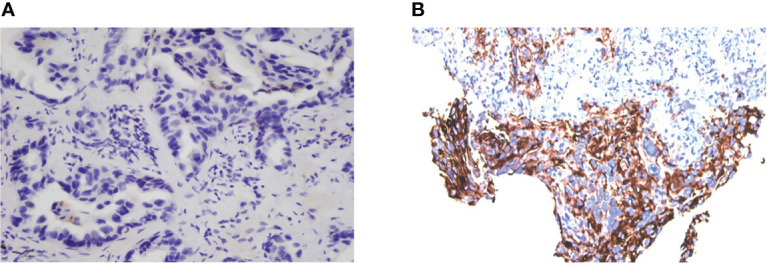
Evaluation of PD-L1 expression of tumor cells. PD-L1 expression of Case 1 in November 2018 **(A)**, TPS = 0 and July 2020 **(B)**, TPS = 60% (Dako 22C3).

In our cases, the PD-L1 expression level increased a lot after EGFR-TKI treatment resistance (**Figures 3A, B**), and a favorable response to subsequent immunotherapy was achieved, as reported by others (7). Because NSCLC patients with EGFR mutation naïve for EGFR-TKI cannot benefit from immunotherapy, the efficacy of immunotherapy could be influenced by tumor microenvironment (TME) changes during the EGFR-TKI treatment (8). Therefore, EGFR-TKIs and immunotherapy may have potential synergistic effects.

The mechanisms of efficacy for furmonertinib or osimertinib rechallenge may be attributed to higher penetration into the CSF (9), recovery of TKI sensitive tumor clones (10), and the histological heterogeneity of intracranial lesions.

However, the precise mechanism of EGFR-TKI rechallenge after immunotherapy failure is unclear. We speculate that two kinds of cell clones may coexist in our cases: one is the EGFR mutant clone (the abundance of clones decreased significantly after TKI treatment, the inferior clone), and the other is the PD-L1 high expression clone (the adaptive production or increase after TKI treatment, the dominant clone). After the immunotherapy, the PD-L1 high-expression clone was at a disadvantage due to its sensitivity to immunotherapy, while the EGFR mutant clone was insensitive to or resistant to immune checkpoint inhibitors. Therefore, the EGFR mutant clone became the dominant clone and then transferred to the leptomeninges (cerebrospinal fluid/blood NGS after immunotherapy resistance also confirmed a significant increase in EGFR abundance), at which time the sensitivity to EGFR-TKI may be restored. So, the EGFR-TKI rechallenge could be effective after immunotherapy failure.

Though irAE did not occur in our cases, the potential toxicity of sequential ICI followed by osimertinib should be monitored closely (11). More clinical studies are needed to explore effective therapy for advanced NSCLC with LM after multi-line treatments.

## Data availability statement

The original contributions presented in the study are included in the article/supplementary material. Further inquiries can be directed to the corresponding author.

## Ethics statement

The studies involving human participants were reviewed and approved by the Nanjing Chest Hospital, The Affiliated Brain Hospital of Nanjing Medical University. Written informed consent for participation was not required for this study in accordance with the national legislation and the institutional requirements. Written informed consent was obtained from the individual(s) for the publication of any potentially identifiable images or data included in this article.

## Author contributions

CQ and SF designed the study. YZ collected the clinical data. QZ and ML participated in collecting the NGS data. YZ and WC analyzed the data. SF reviewed and analyzed data. CQ, YZ, and SF drafted the manuscript. SF supervised the entire study. All authors contributed to the article and approved the submitted version.

## Funding

This work was supported by the “Six One Projects” in Jiangsu Province (LGY2019006). We are thankful to all referring surgeons, pathologists, and specialists for their contributions to this study.

## Acknowledgments

We would like to thank the patients and their families who gave us consent to publish these cases.

## Conflict of interest

Authors QZ and ML were employed by MyGene Diagnostics Co., Ltd.

The remaining authors declare that the research was conducted in the absence of any commercial or financial relationships that could be construed as a potential conflict of interest.

## Publisher’s note

All claims expressed in this article are solely those of the authors and do not necessarily represent those of their affiliated organizations, or those of the publisher, the editors and the reviewers. Any product that may be evaluated in this article, or claim that may be made by its manufacturer, is not guaranteed or endorsed by the publisher.
